# Patient recalls associated with resident-to-attending radiology report discrepancies: predictive factors for risky discrepancies

**DOI:** 10.1186/s13244-022-01233-4

**Published:** 2022-06-04

**Authors:** A Yeon Son, Gil-Sun Hong, Choong Wook Lee, Ju Hee Lee, Won Jung Chung, Jung Bok Lee

**Affiliations:** 1 Department of Radiology and Research Institute of Radiology, University of Ulsan College of Medicine, Asan Medical Center, 88 Olympic-ro 43-gil, Songpa-gu, Seoul, 05505 Republic of Korea; 2 Department of Health Screening and Promotion Center, University of Ulsan College of Medicine, Asan Medical Center, Seoul, Republic of Korea; 3 Department of Clinical Epidemiology and Biostatistics, Asan Medical Center, Seoul, Republic of Korea

**Keywords:** Radiology report discrepancies, Hospitalization, Adverse outcomes, Risk factors

## Abstract

**Background:**

This study aimed to identify predictive factors for risky discrepancies in the emergency department (ED) by analyzing patient recalls associated with resident-to-attending radiology report discrepancies (RRDs).

**Results:**

This retrospective study analyzed 759 RRDs in computed tomography (CT) and magnetic resonance imaging and their outcomes from 2013 to 2021. After excluding 73 patients lost to follow-up, we included 686 records in the final analysis. Risky discrepancies were defined as RRDs resulting in (1) inpatient management (hospitalization) and (2) adverse outcomes (delayed operations, 30-day in-hospital mortality, or intensive care unit admission). Predictors of risky discrepancies were assessed using multivariable logistic regression analysis. The overall RRD rate was 0.4% (759 of 171,419). Of 686 eligible patients, 21.4% (147 of 686) received inpatient management, and 6.0% (41 of 686) experienced adverse outcomes. RRDs with neurological diseases were associated with the highest ED revisit rate (79.4%, 81 of 102) but not with risky RRDs. Predictive factors of inpatient management were critical finding (odds ratio [OR], 5.60; *p* < 0.001), CT examination (OR, 3.93; *p* = 0.01), digestive diseases (OR, 2.54; *p* < 0.001), and late finalized report (OR, 1.65; *p* = 0.02). Digestive diseases (OR, 6.14; *p* = 0.006) were identified as the only significant predictor of adverse outcomes.

**Conclusions:**

Risky RRDs were associated with several factors, including CT examination, digestive diseases, and late finalized reports, as well as critical image findings. This knowledge could aid in determining the priority of discrepancies for the appropriate management of RRDs.

**Supplementary Information:**

The online version contains supplementary material available at 10.1186/s13244-022-01233-4.

## Key points

- Resident-to-attending radiology report discrepancies (RRDs) affected patients negatively despite their rarity.
- Neurological diseases contributed to not risky RRDs but the highest revisit.
- Risky RRDs were associated with CT, digestive diseases, and critical findings.
- Late finalized reports also significantly contributed to risky RRDs.
- Prediction of risky RRDs could provide efficient strategies for managing RRDs.

## Introduction

In recent decades, emergency department (ED) radiology services around the world have moved toward in-house attending coverage for 24 h a day. Although there is growing agreement regarding the necessity of in-house attending radiologists, this service has not achieved universal implementation for a variety of reasons, including the associated costs and reluctance among radiology trainees and attending radiologists [1]. As a result, radiology residents still contribute to ED radiology coverage at academic hospitals, particularly during off-duty periods; this is considered an indispensable component of resident training. Moreover, although preliminary radiology reports generated by trainees are potentially associated with a higher risk of diagnostic errors, they are commonly used in making clinical decisions in the ED. Therefore, discrepancies between preliminary reports by radiology residents and final reports by staff radiologists are an ongoing issue in emergency patient care.

There have been numerous studies on resident-to-attending radiology report discrepancies (RRDs). Most studies have focused on discrepancy rates between preliminary and finalized radiology reports and have underscored the diagnostic imaging modalities in which RRDs are frequently found [2–6]. Several studies have emphasized the importance of resident training level and attending radiologists reading after hours [3, 7]. Relatively few studies have evaluated the clinical impact and costs of RRD-induced patient recalls to the ED; however, many have highlighted the incidence of clinical events, such as changes in patient management or disposition, adverse outcomes, and mortality [7–12]. These prior studies were valuable in that they provided evaluations of the feasibility of continuing the current practices of diagnostic radiology service systems in the ED. However, to our knowledge, there are no published systemic analyses of predictive factors for risky RRDs. Given the limited resources in the ED, it is important to evaluate the discrepancy risk and then address RRDs according to priority.

This study aimed to identify predictive factors for risky RRDs, leading to inpatient management (i.e., admission to hospital) and major adverse outcomes (delayed operations, 30-day in-hospital mortality, or intensive care unit [ICU] admission) by analyzing patient recalls associated with RRDs on computed tomography (CT) and magnetic resonance imaging (MRI).

## Methods

### Study population

**Fig. 1:**
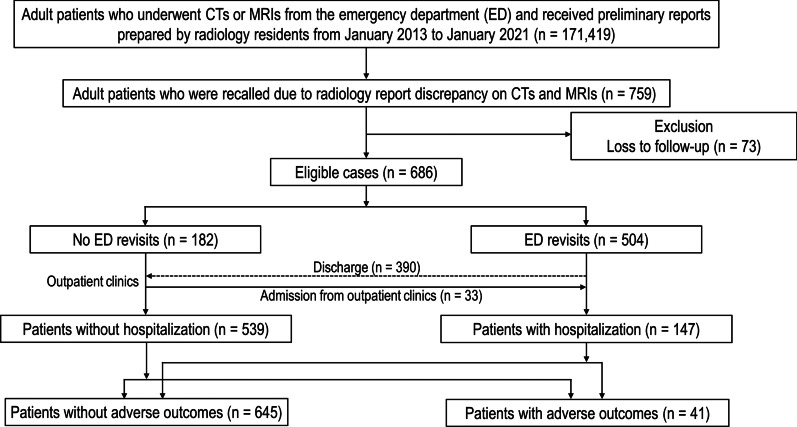
Patient enrollment flowchart. *ED* emergency department, *CT* computed tomography, *MRI* magnetic resonance imaging

This retrospective study was conducted at a tertiary academic hospital that manages > 150,000 ED cases per year. This study included a total of 759 adult patients (≥ 18 years old) who were recalled after discharge from index ED visits due to the discrepancies between the preliminary CT and MRI reports by radiology residents and the final reports by attending radiologists from January 2013 to January 2021. The RRD information was collected from our institution’s Radiology Critical Value Reporting System database, which contains the radiology medical records summarizing patient recalls associated with reading errors for critical or minor findings. Of 759 tests, 73 cases were excluded because of loss to follow-up. Finally, 686 eligible cases were included in this study. Patient recalls were defined as physicians' phone calls to request the patient return to the ED or explain the necessity of short-term follow-up in outpatient clinics due to RRD. However, some patients refused to return to the ED and visited outpatient clinics despite patient recalls. Patient revisits were defined as a patient’s return to the ED in response to patient recalls. Figure 1 illustrates the study enrollment process. Our study did not include pediatric RRDs because our pediatric ED has different radiology coverage systems (including for in-house attending coverage time). Our pediatric ED operates separately from the adult ED and is primarily covered by pediatric radiologists, not emergency radiologists with shift work.

### Data collection

The variables collected were gender, age, radiology examination, preliminary radiology report, final radiology report, duty periods in radiology, time interval between preliminary and final radiology reports, disease categories of final diagnosis, disposition of patients, and adverse outcomes (delayed operations, 30-day in-hospital mortality, or ICU admission). The reading errors for RRDs were analyzed using a modified classification schema by referring to existing studies [13, 14] and then largely classifying errors into two categories (misdiagnosis and perception errors) for statistical analysis. Duty periods were divided into on-duty and off-duty periods according to the presence or absence of in-house attending radiologists. Time intervals between preliminary and final radiology reports were divided into two categories (early finalized report [≤ 9 h] vs. late finalized report [> 9 h]) according to the median time interval determined in our study. The disease categories were classified according to guidelines from the 11th edition of the International Classification of Diseases [15]. The imaging findings in discrepant cases were classified as critical or minor findings. Critical findings were defined as any radiologic finding relevant to the patient’s symptoms that had an immediate impact on patient care and for which an emergency or urgent intervention or response was required.

### Outcome measures

The RRDs were categorized on a scale from 1‒4 according to the patients’ disposition and outcomes [2, 16]. Category 1 was a minor discrepancy with no additional immediate action needed. Category 2 required patients to return to the ED for additional management or symptom control. Category 3 required patients to return to the ED for hospital admission and active treatment. Category 4 denoted a serious risk to patients (i.e., leading to one of the following: delayed operation, 30-day in-hospital mortality, or ICU admission). Category 3 included direct admission from the ED to the hospital and admissions from outpatient follow-up clinics after ED revisits to the hospital. The adverse outcomes defining category 4 were determined by referring to published literature, wherein adverse outcomes are defined as suboptimal patient experiences [16, 17]. In the case of patients being followed up by an outpatient clinic, electronic medical records were evaluated and analyzed to determine whether adverse or major adverse outcomes occurred (> 30 days). The primary outcome was risky discrepancies (risky RRDs), defined as RRDs resulting in (1) inpatient management (category 3) and (2) adverse outcomes (category 4).

### Emergency radiology service system and patient recall system

In January 2011, our institution established a dedicated emergency radiology section. Dedicated radiology residents and dedicated in-house attending radiologists provided emergency radiology services during extended working hours (8:00 am to 10:00 pm on weekdays and 1:00 pm to 9:00 pm on weekends and holidays). Since March 2019, the in-house emergency radiology attending service has further extended the ED coverage hours (8:00 am to 10:00 pm on weekdays and 9:00 am to 9:00 pm on weekends and holidays). Outside of the working hours above, senior radiology residents provide preliminary reports to the referring clinicians at the ED with an available subspecialty faculty backup. Attending radiologists then review the preliminary reports and sign off the final reports the following day. To avoid delayed notifications or missed follow-ups, our patient recall system includes a streamlined reporting system. For critical discrepancies, attending radiologists refer discrepant cases to a discrepant liaison physician through a single phone line and then report the discrepant cases using the Critical Value Reporting System, which automatically sends a text message to notify on-call physicians. For minor discrepancies, attending radiologists only report discrepant cases using the Critical Value Reporting System. All discrepancies were notified to the affected patients by phone, with further explanations regarding the need for patients to return to the ED or attend outpatient clinics.

### Statistical analysis

Comparisons between each of the discrepant category 3 or 4 groups and the control group were performed using the Chi-square test and Fisher's exact test for categorical variables and Student's *t* test and the Mann–Whitney *U* test for continuous variables. Univariable and multivariable logistic regression analyses were performed with the stepwise method using penalized maximum likelihood estimation to identify independent predictive factors for risky RRDs (inpatient management [discrepant category 3] or adverse outcomes [discrepant category 4]). All statistical analyses were performed using SPSS Statistics for Windows (version 21, IBM Corp., Armonk, NY, USA) and SAS (version 9.4, SAS Institute, Cary, NC, USA); *p* values < 0.05 were considered to indicate statistical significance.

## Results

### Patient characteristics

Table 1 summarizes the patient characteristics. During the study period, 0.4% (759 of 171,419) of ED patients were recalled due to RRDs on CTs or MRIs. Among 686 eligible patients (mean age ± standard deviation [SD], 55.3 years ± 18; 356 females [51.9%]), 79.3% (544 of 686) received recalls due to discrepancies in critical radiologic findings. The leading cause of RRDs in terms of diagnostic errors was perception error (75.7%, 519 of 686) (see Additional file 1: Table S1 for details of the causes of diagnostic errors in discrepancies). The most common disease category associated with RRDs was neoplasms (26.2%, 180 of 686), followed by digestive diseases (21.9%, 150 of 686) and neurologic diseases (14.9%, 102 of 686) (see Additional file 1: Table S2 for a summary of the actual pathologic diseases associated with RRDs). In terms of diagnostic imaging modalities, RRDs were most frequently detected in abdominal pelvic CT (47.8%, 328 of 686), followed by head CT (19.1%, 131 of 686). RRDs occurred more frequently in off-duty periods (60.8%, 417 of 686). More than half of the cases (58.5%, 401 of 686) had a late finalized report (> 9 h).

**Table 1:** Baseline characteristics of study sample

Variables	
Total patients	N = 686
Sex, female^a^	356 (51.9%)
Age (mean ± SD years)^b^	55.3 ± 18
Categories of diagnostic errors^a^	
Perception error	519 (75.7%)
Misdiagnosis	167 (24.3%)
Categories of diseases^a^	
Neoplasm	180 (26.2%)
Digestive	150 (21.9%)
Neurologic	102 (14.9%)
Trauma	76 (11.1%)
Genitourinary	36 (5.2%)
Others	142 (20.7%)
Image findings^a^	
Minor findings	142 (20.7%)
Critical findings	544 (79.3%)
Inpatient management	147 (21.4%)
Adverse outcomes^a^	41 (6.0%)
Conditions	
(1) Delayed operation	40 (5.8%)
(2) 30-day in-hospital mortality	0 (0.0%)
(3) ICU admission	1 (0.1%)
Examination^a^	
CT abdomen and pelvis	328 (47.8%)
CT head	131 (19.1%)
CT vessel	65 (9.5%)
CT chest	48 (7.0%)
CT whole body trauma	20 (2.9%)
CT neck	18 (2.6%)
CT musculoskeletal	3 (0.4%)
CT spines	1 (0.1%)
MRI head	66 (9.6%)
MRI spine	6 (0.9%)
Duty periods^a^	
On-duty period	269 (39.2%)
Off-duty period	417 (60.8%)
Time internal between preliminary and finalized reports^a^	
Early finalized reports (≤ 9 h)	285 (41.5%)
Late finalized reports (> 9 h)	401 (58.5%)

^a^Data are number of patients, with percentages in parentheses. ^b^Data are mean ± standard deviation. The sum of percentages may not be exactly 100% owing to rounding

*CT* Computed tomography, *ICU* intensive care unit, *MRI* magnetic resonance imaging

### RRD categories according to disease categories and radiologic factors

**Fig. 2:**
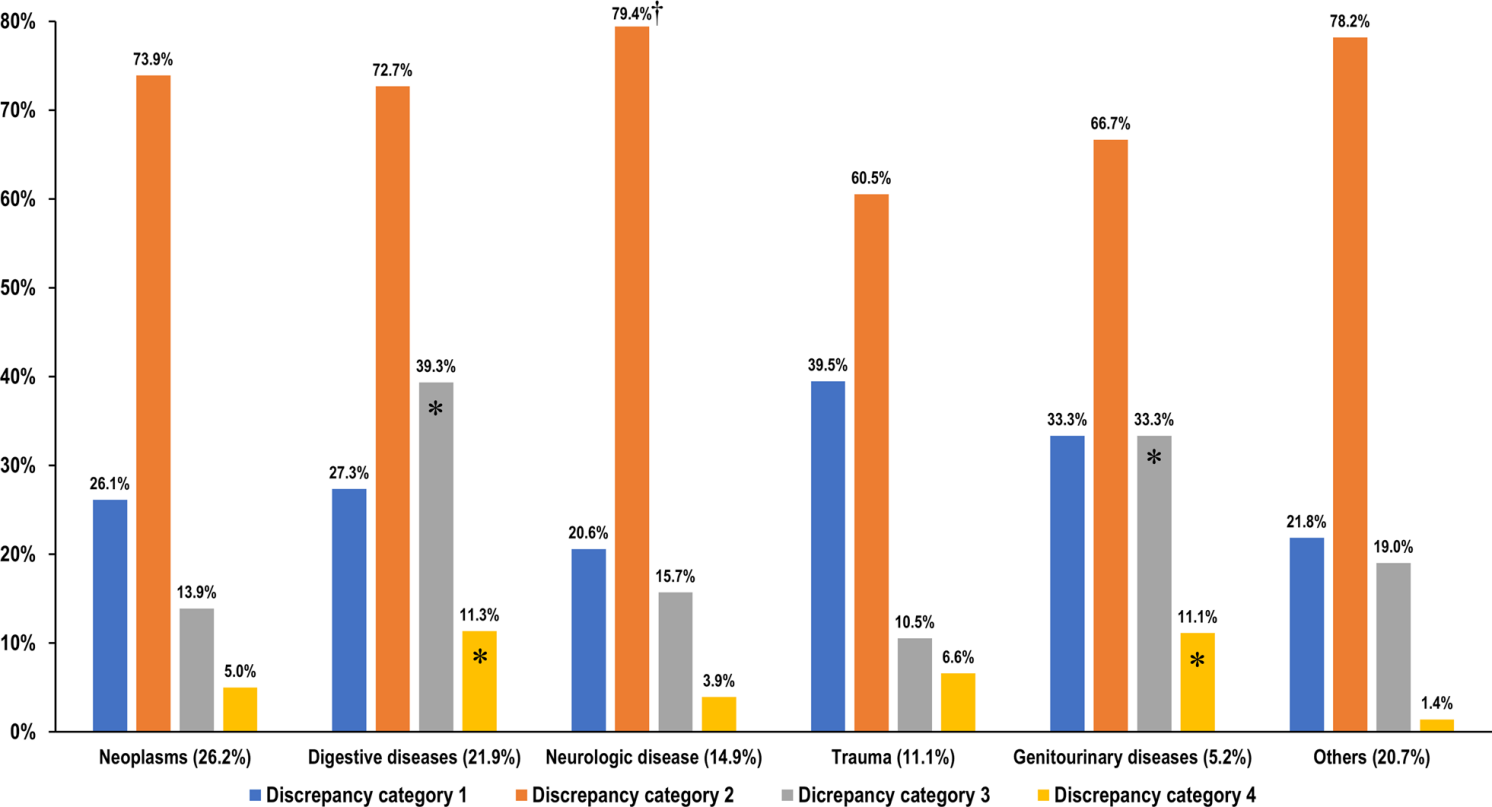
Incidence of discrepancy categories according to disease category. *Each of discrepancy category 3 (inpatient management) and category 4 (adverse outcomes) occurred most frequently in association with digestive diseases, followed by genitourinary diseases. ^†^Neurologic diseases were associated with the highest emergency department revisit rate

Of the discrepant cases, 26.5% (182 of 686) were in category 1, 73.5% (504 of 686) were in category 2, 21.4% (147 of 686) were in category 3, and 6.0% (41 of 686) were in category 4. Figure 2 summarizes the incidence of each RRD category according to disease category. Of patient recalls with RRDs, our results revealed a wide range of ED revisit rates, from about 60% to 80%, depending on the disease category. Neurologic diseases were associated with the highest rate of ED revisits (79.4%, 81 of 102) but low rates of inpatient management and adverse outcomes (15.7% [16 of 102] and 3.9% [4 of 102], respectively). Although the neoplasm category occupied the most significant proportion of patient recalls, the rates of inpatient management and adverse outcomes were relatively low (13.9% [25 of 180] and 5.0% [9 of 180], respectively). The highest rates of inpatient management and adverse outcomes occurred in digestive diseases (39.3% [59 of 150] and 11.3% [17 of 150], respectively), followed by genitourinary diseases (33.3% [12 of 36] and 11.1% [4 of 36], respectively).

**Fig. 3:**
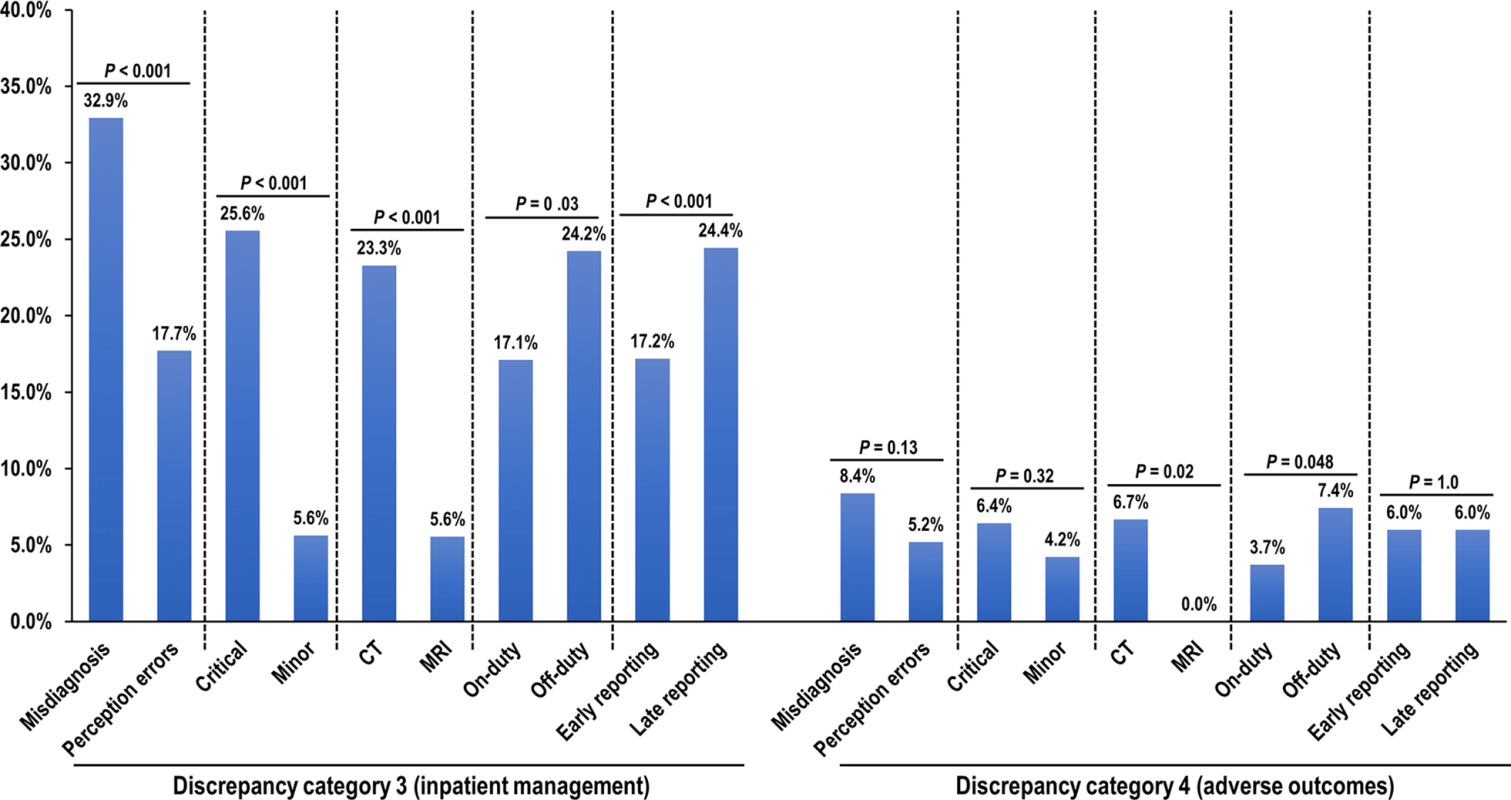
Incidence of discrepancy category 3 (inpatient management) and category 4 (adverse outcomes) according to various factors associated with radiologic diagnosis. *CT* computed tomography, *MRI* magnetic resonance imaging

Figure 3 illustrates the rates of inpatient management and adverse outcomes according to the various radiologic factors. Inpatient management rates were significantly higher in the following conditions: misdiagnosis than perception errors (32.9% [55 of 167] vs. 17.7% [92 of 519], *p* < 0.001); critical findings than minor findings (25.6% [139 of 544] vs. 5.6% [8 of 142], *p* < 0.001); CT than MRI (23.3% [143 of 614] vs. 5.6% [4 of 72], *p* < 0.001); off-duty periods than on-duty periods (24.2% [101 of 417] vs. 17.1% [46 of 269], *p* = 0.03); and late finalized reports than early finalized reports (24.4% [98 of 401] vs. 17.2% [49 of 285], *p* < 0.001). The adverse outcome rate did not significantly differ according to the diagnostic error type, the presence or absence of critical findings, and the time interval between preliminary and finalized reports. However, the adverse outcome rate was significantly higher in the following conditions: CT than MRI (6.7% [41 of 614] vs. 0.0% [0 of 72], *p* = 0.02) and off-duty periods than on-duty periods (7.4% [31 of 417] vs. 3.7% [10 of 269], *p* = 0.048).

### Predictive factors for risky RRDs

Table 2 shows the results of the univariable and multivariable logistic regression analyses for risky RRDs. Multivariable logistic regression analysis revealed the following predictors of inpatient management (category 3): critical finding (odds ratio [OR], 5.60; *p* < 0.001), CT (OR, 3.93; *p* = 0.01), digestive diseases (OR, 2.54; *p* < 0.001), and late finalized report (OR, 1.65; *p* = 0.02). Table 3 summarizes predictors of adverse outcomes (category 4). Digestive diseases (OR 6.14; *p* = 0.006) were identified as the only significant predictive factor.

**Table 2:** Univariable and multivariable logistic regression analysis of predictive factors for inpatient management

Variables	Univariable analysis		Multivariable analysis	
	OR (95% CI)	*p* value	OR (95% CI)	*p* value
*Age*				
Gender (male)	1.00 (0.99–1.01)	0.34		
Male	0.94 (0.65–1.30)	0.75		
Female	1.00			
Error category				
Misdiagnosis	2.28 (1.54–3.38)	< 0.001		
Perception	1.0			
Disease categories				
Neoplasm	0.69 (0.38–1.25)	0.03	1.40 (0.73–2.68)	0.68
Digestive	2.76 (1.62–4.70)	< 0.001	2.54 (1.47–4.36)	< 0.001
Neurologic	0.79 (0.40–1.56)	0.21	1.20 (0.59–2.47)	0.85
Trauma	0.50 (0.22–1.17)	0.02	0.46 (0.20–1.08)	0.002
Genitourinary	2.13 (0.95–4.79)	0.03	2.08 (0.91–4.76)	0.12
Others	1.00		1.00	
Critical findings				
Critical	5.75 (2.75–12.04)	< 0.001	5.60 (2.48–12.64)	< 0.001
Minor	1.00		1.00	
Imaging modality				
CT	5.16 (1.85–14.39)	0.002	3.93 (1.34–11.54)	0.01
MRI	1.00		1.00	
Duty periods				
Off-duty period	1.55 (1.05–2.29)	0.03		
On-duty period	1.00			
Time interval between preliminary and finalized reports				
Late finalized reports (> 9 h)	1.56 (1.06–2.28)	0.02	1.65 (1.10–2.47)	0.02
Early finalized reports (≤ 9 h)	1.00		1.00	

Data are shown as odds ratios with 95% confidence intervals in parentheses

*CT* computed tomography, *CI* confidence interval, *MRI* magnetic resonance imaging, *OR* odds ratio

**Table 3:** Univariable and multivariable logistic regression analysis of predictive factors for adverse outcomes

Variables	Univariable analysis		Multivariable analysis	
	OR (95% CI)	*p* value	OR (95% CI)	*p* value
Age				
Age	1.00 (0.98–1.01)	0.71		
Gender				
Male	0.93 (0.49–1.75)	0.82		
Female				
Error category				
Misdiagnosis	1.67 (0.85–3.26)	0.14		
Perception	1.0			
Disease categories				
Neoplasm	2.70 (0.72–10.20)	0.91	2.70 (0.72–10.20)	0.91
Digestive	6.14 (1.76–21.28)	0.006	6.14 (1.76–21.28)	0.006
Neurologic	1.49 (0.29–7.52)	0.22	1.49 (0.29–7.52)	0.22
Trauma	3.47 (0.81–14.93)	0.62	3.47 (0.81–14.93)	0.62
Genitourinary	5.71 (1.22–27.03)	0.13	5.71 (1.22–27.03)	0.13
Others	1.00			
Critical findings				
Critical	1.56 (0.64–3.79)	0.33		
Minor	1.00			
Imaging modality				
CT	10.53 (0.63–166.67)	0.10		
MRI	1.00			
Duty periods				
Off-duty period				
On-duty period	2.08 (1.002–4.31)	0.05		
Time interval between preliminary and finalized reports				
Late finalized reports (> 9 h)	1.00 (0.53–1.91)	0.99		
Early finalized reports (≤ 9 h)	1.00			

Data are shown as odds ratios with 95% confidence intervals in parentheses

*CT* computed tomography, *CI* confidence interval, *MRI* magnetic resonance imaging, *OR* odds ratio

## Discussion

Our study was the first to identify predictors of risky discrepancies necessitating inpatient management and closer monitoring of adverse outcome risk. To date, most previous studies have focused on low discrepancy rates with minor negative effects on patient safety. Although these results support the rationale of residents’ preliminary interpretations in academic hospitals, previous studies have been limited in terms of their ability to provide information on the characteristics of risky discrepancies or the severity of such discrepancies.

Regarding the clinical impact of RRDs, our results were comparable to those in previous studies. In the present study, RRDs led to inpatient management (21.4%) and adverse outcomes (6.0%); however, there was no 30-day in-hospital mortality. Carney et al. [18] reported a major discrepancy rate (1.0%) on body CTs leading to changes in patient management but without a negative impact on patient morbidity. Chung et al. [19] noted a major discrepancy rate (0.3%) among abdominopelvic CTs associated with changes in patient management. Lal et al. [20] reported that 0.08% of discrepant neuroradiological CT scans had potentially serious negative effects on patient outcomes. Ruchman et al. [7] reported that 6.9% of RRDs had a negative impact on patient care, and 0.3% had significantly negative effects. In emergency medicine, a previous study reported a relatively high radiology discrepancy rate (57.1%) with a major clinical impact; however, that study was limited by its small sample size (*n* = 28) [8].

Our data suggest that risky discrepancies have several distinct features from the overall discrepancy group. In the overall discordant population, results from our study were similar to those of previous studies. Our overall discrepancy rate (0.4%) was consistent with those found in previous studies of resident-to-attending discrepancies (ranging from 0.2 to 3.8%) [7, 18, 21–25]. Friedman et al. [8] reported that discrepancies occurred most frequently in association with abdominopelvic CTs (32.1%), followed by head CTs (25%). Buchman et al. [7] similarly found abdominopelvic CT scans to be the most common source of discordance between residents and attending physicians (56.4%). These findings were comparable to ours. In line with the study by Jeong et al. [3], our discrepant cases occurred more commonly during off-duty periods. The leading diagnostic error of discrepancies in our study was perception error, and major diseases were neoplasm, digestive diseases, and neurologic diseases. These results aligned well with those reported in the published literature on diagnostic errors in radiology [14, 26–30]. Notably, in the risky RRD group, the major disease category was digestive diseases, not neoplasms and neurologic diseases. The leading cause of risky discrepancies was misdiagnosis, and not perception error. This may be because most perceptual errors are associated with mild disease with subtle image detection. So, if ED revisits occur within a short period, the disease may not have progressed to a serious condition. In contrast, most misdiagnosis occurs in association with cases with imaging findings that are readily detectable but misinterpreted by residents. Therefore, they could be more advanced disease states. In addition, misdiagnoses can cause poor management and misguided discharge orders. Therefore, patients returning to the ED may be more likely to have severe condition and require hospitalization and surgery (e.g., if acute appendicitis is mistaken for diverticulitis). Interestingly, the late finalized report rate was significantly higher than the early finalized report rate in the risky RRD group.

Our study identified the predictors of risky RRDs as follows: critical findings, CT examination, digestive diseases, and late finalized reports. Our results are supported by those in previous studies of diagnostic errors. Carrara et al. [31] reported that abdominal disease was the most common disease (44.1%) associated with diagnostic CT and MRI errors. Chang et al. [32] found that digestive diseases were common causes of ED revisits and ICU admissions. Interestingly, despite being associated with the highest ED revisit rate in our study, neurologic diseases were not predictors of risky RRDs. This might have been because this category mainly consisted of patients with small amount of traumatic intracranial hemorrhages on brain CT scans; these usually do not need aggressive management because of the low risk of rapid deterioration. The neoplasm category likely accounted for the largest proportion of discrepancies but was not identified as predictive of risky RRDs. This was because most patients in this category received symptom management in the ED and were followed up as outpatients. Notably, in our study, late finalized reports were critical predictive factors for risky RRDs. The benefits of faster turnaround times in radiology are well known. Late finalized reports are reasonably expected to lead to delayed notifications to ED physicians and patients, as well as delayed clinical treatment. In other words, the longer the delay in releasing a finalized report, the more likely it is for a mild illness to progress to a more severe condition. For example, patients with mild diverticulitis can be treated in an outpatient setting if they receive appropriate and timely medical care in the ED. However, in the case of a delayed recall, mild diverticulitis may progress to a severe state (e.g., systemic infection, peritonitis, and perforation) requiring hospitalization and surgical treatment. Dabbo et al. [33] reported that reduced turnaround times for finalized CT reports led to significant reductions in the amount of time (up to 7 h sooner) that ED physicians had to resolve discrepant reports. Our findings may help improve patient care by identifying risky discrepancies and facilitating earlier resolutions to discrepancy-related problems.

This study had several limitations. First, as a study conducted at a single tertiary hospital, there may be limitations in its generalizability. Second, the resident-to-attending report discrepancies were evaluated based on the final attending reports; however, the accuracy of final attending reports was not evaluated. Nevertheless, it reflects the real-world process because, in actual practice, patient recalls are decided based on the final radiology reports prepared by attending radiologists. Third, the discrepant cases could have been reviewed by available faculty backup during off-duty periods. However, the retrospective study design over a long period could have limited the evaluation of certain details in this regard. Fourth, outcomes measured in the present study heavily relied on unmeasurable variables (e.g., emergency care system, health costs, and health insurance system); however, the effects of these variables are difficult to define because of their complexity. Fifth, selection bias may have played a role in this study because the criteria for patient recalls may vary depending on the individual characteristics of radiologists or ED physicians. Particularly, minor discrepant cases could be overlooked in a busy emergency department, which would lead to an underestimated discrepancy rate. Finally, there is no consensus about risky discrepancies and the extent of their negative impact on patient care. The relevance of discrepancies to patient care and the negative impact has been measured variously in previous studies. To mitigate this bias, we investigated only inpatient management rates and adverse outcome rates for assessing effects on patient care; this, however, could have led to underestimates in our results.

## Conclusions

Our study identified predictors of risky discrepancies in radiology, leading to inpatient management and adverse outcomes. Although neoplasms and neurologic diseases accounted for a large proportion of our discrepant cases, they had little contribution to risky discrepancies. Our findings suggest that radiologists and ED physicians should pay attention to discrepancies in critical findings, particularly when CT is used, especially in the diagnosis and management of digestive diseases. Our results also revealed an association between late finalized reports and hospital admission. This finding highlights the importance of reducing the time from preliminary to finalized reporting. Although patient characteristics and conditions are most critical when making clinical decisions in light of RRDs, radiologists in the ED often have inadequate clinical information at the time of interpretation. Therefore, from the radiologist’s perspective, these predictive factors are important for assessing the level of risk associated with RRDs. The present study provides information on the priority of discrepancies and the necessity of patient recalls, which is vital for determining the appropriate management steps for RRDs and for facilitating communication among physicians to prevent delays in care and patient notification, as well as to prevent patients from being lost to follow-up.

## Supplementary Information

**Additional file 1.** The causes of diagnostic errors contributing to discrepancies and a list of actual pathologic diseases associated with discrepancies according to the patient disposition, management, and adverse outcomes.

## Data Availability

The datasets used and/or analyzed for this study are available from the corresponding author on reasonable request.

## Abbreviations

CI: Confidence interval
CT: Computed tomography
ED: Emergency department
ICU: Intensive care unit
MRI: Magnetic resonance imaging
OR: Odds ratio
RRDs: Resident-to-attending radiology report discrepancies
