# Supplementary material for: Patient recalls associated with resident-to-attending radiology report discrepancies: predictive factors for risky discrepancies
Source: Insights Imaging. 2022 Jun 4;13:97. doi: 10.1186/s13244-022-01233-4 (PMC9167364; doi:10.1186/s13244-022-01233-4)
Supplement: Supplementary file 1 — Additional file 1. The causes of diagnostic errors contributing to discrepancies and a list of actual pathologic diseases associated with discrepancies according to the patient disposition, management, and adverse outcomes. [file 13244_2022_1233_MOESM1_ESM.docx]

**ELECTRONIC SUPPLEMENTARY MATERIAL**

**Patient recalls associated with resident-to-attending radiology report discrepancies: predictive factors for risky discrepancies**

**The causes of diagnostic errors contributing to discrepancies and a list of actual pathologic diseases associated with discrepancies according to the patient disposition, management, and adverse outcomes**

**Supplementary Table 1** Categorization of the causes of diagnostic errors contributing to resident-to-attending radiology report discrepancies

| **Type** | **Cause of error** | **Explanation** | **Total** | **Grade 3** | **Grade 4** |
| --- | --- | --- | --- | --- | --- |
| 1 | Complacency | Error of overreading and misinterpretation, in which a finding is appreciated but is attributed to the wrong cause. | 20 | 8 | 1 |
| 2 | Faulty reasoning | Error of overreading and misinterpretation, in which a finding is identified and interpreted as abnormal but is attributed to the wrong cause. Misleading information and a limited differential diagnosis are included in this category. | 129 | 39 | 9 |
| 3 | Lack of knowledge | The finding is seen but is attributed to the wrong cause because of a lack of knowledge on the part of the viewer or interpreter. | 18 | 8 | 4 |
| 4 | Underreading | The finding is missed. | 490 | 89 | 26 |
| 5 | Poor communication | The lesion is identified and interpreted correctly, but the message fails to reach the clinician. | 0 | 0 | 0 |
| 6 | Technique | The finding is missed because of the limitations of the examination or technique. | 1 | 0 | 0 |
| 7 | Prior examination | The finding is missed because of failure to consult prior to radiologic studies or reports. | 0 | 0 | 0 |
| 8 | History | The finding is missed because of the acquisition of inaccurate or incomplete clinical history. | 0 | 0 | 0 |
| 9 | Location | The finding is missed because of the location of a lesion outside the area of interest on an image, such as in the corner of an image. | 28 | 3 | 1 |
| 10 | Satisfaction of search | The finding is missed because of failure to continue to search for additional abnormalities after the first abnormality was found. | 0 | 0 | 0 |
| 11 | Satisfaction of report | The finding was missed due to complacency and overreliance on the radiology report of the previous examinations. | 0 | 0 | 0 |

Data are number of patients applicable to each item. This categorization system is a modification of the classification schemes proposed by Kim and Mansfield [1].

**References**

1. Kim YW, Mansfield LT (2014) Fool me twice: delayed diagnoses in radiology with emphasis on perpetuated errors. AJR Am J Roentgenol 202:465-470 <https://doi.org/10.2214/AJR.13.11493>

**Supplementary Table 2** List of actual pathologic diseases associated with discrepancies according to the patient disposition, management, and adverse outcomes

|  | No revisit | Revisit and discharge | Revisit with admission | Delayed operation or ICU admission | Total |
| --- | --- | --- | --- | --- | --- |
| **Neoplasms** | **47** | **108** | **16** | **9** | **180** |
| Lung cancer | 7 | 16 | 3 | 0 | 26 |
| Intracranial tumor | 3 | 15 | 0 | 1 | 19 |
| Small bowel tumor | 3 | 9 | 0 | 1 | 13 |
| Bone tumor | 2 | 9 | 0 | 1 | 12 |
| Pituitary gland tumor | 3 | 8 | 0 | 1 | 12 |
| Colon cancer | 4 | 4 | 1 | 2 | 11 |
| Breast nodule | 4 | 4 | 0 | 0 | 8 |
| Pancreatic cancer | 3 | 3 | 0 | 0 | 6 |
| Renal cell carcinoma of kidney | 1 | 3 | 1 | 0 | 5 |
| Ureter cancer | 1 | 2 | 2 | 0 | 5 |
| Gastric cancer | 1 | 2 | 0 | 1 | 4 |
| HCC rupture | 0 | 2 | 2 | 0 | 4 |
| Metastasis | 1 | 3 | 0 | 0 | 4 |
| Bladder cancer | 1 | 2 | 0 | 0 | 3 |
| Gallbladder cancer | 1 | 1 | 0 | 1 | 3 |
| Pancreatic neoplasm | 1 | 2 | 0 | 0 | 3 |
| Periampullary tumor | 0 | 2 | 1 | 0 | 3 |
| Prostate cancer | 0 | 2 | 1 | 0 | 3 |
| HCC bile duct invasion | 0 | 0 | 2 | 0 | 2 |
| Hepatic tumor | 0 | 2 | 0 | 0 | 2 |
| Intrahepatic cholangiocarcinoma | 1 | 0 | 0 | 1 | 2 |
| Lung nodule | 1 | 1 | 0 | 0 | 2 |
| Parotid gland tumor | 0 | 2 | 0 | 0 | 2 |
| Retroperitoneal mass | 1 | 1 | 0 | 0 | 2 |
| Salivary gland tumor | 1 | 1 | 0 | 0 | 2 |
| AOV cancer | 0 | 0 | 1 | 0 | 1 |
| AOV tumor | 0 | 1 | 0 | 0 | 1 |
| Benign neoplasm of ovary | 0 | 1 | 0 | 0 | 1 |
| Bile duct cancer | 0 | 1 | 0 | 0 | 1 |
| Cardiac mass | 1 | 0 | 0 | 0 | 1 |
| Cervical cancer | 0 | 1 | 0 | 0 | 1 |
| Gastric benign tumor | 1 | 0 | 0 | 0 | 1 |
| HCC internal hemorrhage | 1 | 0 | 0 | 0 | 1 |
| Hepatocellular carcinoma | 1 | 0 | 0 | 0 | 1 |
| Intraductal papillary neoplasm | 0 | 1 | 0 | 0 | 1 |
| Leptomeningeal metastasis | 0 | 1 | 0 | 0 | 1 |
| Mediastinal tumor | 0 | 1 | 0 | 0 | 1 |
| Mesentery tumor | 0 | 1 | 0 | 0 | 1 |
| Optic nerve tumor | 1 | 0 | 0 | 0 | 1 |
| Ovarian cancer | 0 | 0 | 1 | 0 | 1 |
| Parotid gland cancer | 1 | 0 | 0 | 0 | 1 |
| Pericardial cyst | 0 | 1 | 0 | 0 | 1 |
| Renal mass | 0 | 1 | 0 | 0 | 1 |
| Sinonasal papilloma, inverted | 1 | 0 | 0 | 0 | 1 |
| Small bowel cancer | 0 | 0 | 1 | 0 | 1 |
| Spinal cord mass | 0 | 1 | 0 | 0 | 1 |
| Tumor in head and neck | 0 | 1 | 0 | 0 | 1 |
| **Digestive diseases** | **41** | **50** | **42** | **17** | **150** |
| Acute appendicitis | 10 | 6 | 20 | 5 | 41 |
| Acute cholecystitis | 4 | 8 | 5 | 4 | 21 |
| Bowel obstruction | 4 | 8 | 7 | 1 | 20 |
| Calculus of bile duct | 2 | 4 | 3 | 3 | 12 |
| Acute diverticulitis | 6 | 1 | 0 | 1 | 8 |
| Bowel perforation | 3 | 1 | 0 | 2 | 6 |
| Ischemic colitis | 1 | 2 | 1 | 0 | 4 |
| Acute pancreatitis | 0 | 3 | 0 | 0 | 3 |
| Hepatic abscess | 0 | 1 | 2 | 0 | 3 |
| Marginal ulcer | 1 | 2 | 0 | 0 | 3 |
| Colitis | 0 | 1 | 1 | 0 | 2 |
| Enteritis | 1 | 1 | 0 | 0 | 2 |
| Gastric ulcer perforation | 0 | 0 | 1 | 1 | 2 |
| Hemoperitoneum | 1 | 1 | 0 | 0 | 2 |
| Internal hernia | 2 | 0 | 0 | 0 | 2 |
| Terminal ileitis | 2 | 0 | 0 | 0 | 2 |
| Bezoar | 0 | 1 | 0 | 0 | 1 |
| Colon stricture | 0 | 1 | 0 | 0 | 1 |
| Dolichocolon | 0 | 1 | 0 | 0 | 1 |
| Gastric ulcer | 0 | 1 | 0 | 0 | 1 |
| Hemobilia | 0 | 1 | 0 | 0 | 1 |
| Hepatic complicated cyst | 0 | 1 | 0 | 0 | 1 |
| Hernia of colon | 0 | 1 | 0 | 0 | 1 |
| Infectious colitis | 0 | 1 | 0 | 0 | 1 |
| Intussusception of small intestine | 1 | 0 | 0 | 0 | 1 |
| Normal bowel | 0 | 1 | 0 | 0 | 1 |
| Perforation of stomach | 0 | 0 | 1 | 0 | 1 |
| Periappendiceal abscess | 1 | 0 | 0 | 0 | 1 |
| Peritonitis | 1 | 0 | 0 | 0 | 1 |
| Pneumatosis intestinalis of large intestine | 0 | 1 | 0 | 0 | 1 |
| Pseudoaneurysm-gastric wall fistula | 0 | 0 | 1 | 0 | 1 |
| Small bowel hematoma | 1 | 0 | 0 | 0 | 1 |
| Tuberculosis of the digestive system | 0 | 1 | 0 | 0 | 1 |
| **Neurologic disease** | **21** | **65** | **12** | **4** | **102** |
| Intracranial hemorrhage | 12 | 42 | 6 | 2 | 62 |
| Cerebral aneurysm, nonruptured | 6 | 9 | 0 | 2 | 17 |
| Cerebral ischemia | 1 | 8 | 4 | 0 | 13 |
| Intracranial vascular malformation | 1 | 1 | 0 | 0 | 2 |
| Cortical dysplasia | 1 | 0 | 0 | 0 | 1 |
| Dural sinus thrombus | 0 | 0 | 1 | 0 | 1 |
| Encephalitis | 0 | 1 | 0 | 0 | 1 |
| Intracranial artery stenosis | 0 | 1 | 0 | 0 | 1 |
| Intracranial vertebral artery dissection | 0 | 0 | 1 | 0 | 1 |
| Moyamoya disease | 0 | 1 | 0 | 0 | 1 |
| RCVS | 0 | 1 | 0 | 0 | 1 |
| Wernicke | 0 | 1 | 0 | 0 | 1 |
| **Traumatic injuries** | **30** | **38** | **3** | **5** | **76** |
| Fracture of skull or facial bones | 12 | 26 | 0 | 5 | 43 |
| Vertebral fracture | 5 | 2 | 1 | 0 | 8 |
| Compression fracture | 1 | 3 | 0 | 0 | 4 |
| Rib fracture | 2 | 2 | 0 | 0 | 4 |
| Sternal fracture | 1 | 2 | 0 | 0 | 3 |
| Lens subluxation | 0 | 1 | 1 | 0 | 2 |
| Pelvic bone fracture | 2 | 0 | 0 | 0 | 2 |
| Clavicular fracture | 1 | 0 | 0 | 0 | 1 |
| Hemorrhage of orbit | 0 | 1 | 0 | 0 | 1 |
| Hemopneumothorax | 1 | 0 | 0 | 0 | 1 |
| Injury of small intestine | 1 | 0 | 0 | 0 | 1 |
| Intracranial artery dissection | 1 | 0 | 0 | 0 | 1 |
| Omental contusion | 0 | 0 | 1 | 0 | 1 |
| Patellar fracture | 0 | 1 | 0 | 0 | 1 |
| Penile injury | 1 | 0 | 0 | 0 | 1 |
| Submandibular gland contusion | 1 | 0 | 0 | 0 | 1 |
| Thyroid cartilage and hyoid bone fracture | 1 | 0 | 0 | 0 | 1 |
| **Genitourinary diseases** | **12** | **12** | **8** | **4** | **36** |
| Ovarian tumor/cyst torsion | 6 | 1 | 2 | 4 | 13 |
| Female pelvic inflammatory diseases | 1 | 1 | 3 | 0 | 5 |
| Urinary tract stone | 1 | 4 | 0 | 0 | 5 |
| Ovarian tumor/cyst rupture | 1 | 1 | 2 | 0 | 4 |
| Renal hematoma | 0 | 2 | 0 | 0 | 2 |
| Abscess of prostate | 0 | 2 | 0 | 0 | 1 |
| Bladder perforation | 0 | 0 | 1 | 0 | 1 |
| Complicated ovarian cyst | 0 | 1 | 0 | 0 | 1 |
| Cystitis | 1 | 0 | 0 | 0 | 1 |
| Hemorrhagic renal cyst | 1 | 0 | 0 | 0 | 1 |
| Injury of urethra | 1 | 0 | 0 | 0 | 1 |
| Malposition of foley catheter | 0 | 1 | 0 | 0 | 1 |
| **Circulatory disease** | **12** | **29** | **16** | **2** | **59** |
| Pulmonary thromboembolism | 5 | 6 | 3 | 0 | 14 |
| Coronary artery stenosis | 0 | 8 | 4 | 1 | 13 |
| Arterial aneurysm or dissection | 1 | 2 | 6 | 0 | 9 |
| Aortic aneurysm or dissection | 2 | 2 | 1 | 0 | 5 |
| Valvular disease | 0 | 3 | 0 | 0 | 3 |
| Active bleeding | 1 | 0 | 1 | 0 | 2 |
| Acute lower limb arterial occlusion | 0 | 0 | 1 | 1 | 2 |
| Thrombosis of mesenteric vein | 0 | 2 | 0 | 0 | 2 |
| Celiac axis occlusion | 1 | 0 | 0 | 0 | 1 |
| Coronary artery anomaly | 0 | 1 | 0 | 0 | 1 |
| Hemopericardium | 1 | 0 | 0 | 0 | 1 |
| ICA stenosis | 0 | 1 | 0 | 0 | 1 |
| LA thrombus | 0 | 1 | 0 | 0 | 1 |
| Pericarditis | 0 | 1 | 0 | 0 | 1 |
| SMA stenosis | 0 | 1 | 0 | 0 | 1 |
| Thrombophlebitis | 0 | 1 | 0 | 0 | 1 |
| Thrombosis of ovarian vein | 1 | 0 | 0 | 0 | 1 |
| **Nondiagnostic** | **8** | **20** | **0** | **0** | **28** |
| **Musculoskeletal or connective tissue diseases** | **5** | **16** | **1** | **0** | **22** |
| Intramuscular hemorrhage | 0 | 4 | 0 | 0 | 4 |
| Arthritis | 1 | 2 | 0 | 0 | 3 |
| Infectious spondylitis | 0 | 3 | 0 | 0 | 3 |
| Infectious myositis | 0 | 2 | 0 | 0 | 2 |
| Myelopathy | 1 | 1 | 0 | 0 | 2 |
| Osteomyelitis | 0 | 2 | 0 | 0 | 2 |
| Disc bulging | 1 | 0 | 0 | 0 | 1 |
| Discitis | 1 | 0 | 0 | 0 | 1 |
| Septic arthritis | 0 | 0 | 1 | 0 | 1 |
| Spondylolisthesis | 1 | 0 | 0 | 0 | 1 |
| Subcutaneous emphysema | 0 | 1 | 0 | 0 | 1 |
| Synovitis | 0 | 1 | 0 | 0 | 1 |
| **Respiratory diseases** | **1** | **5** | **6** | **0** | **12** |
| Tuberculosis of the respiratory system | 0 | 2 | 5 | 0 | 7 |
| Lung AVM | 0 | 1 | 0 | 0 | 1 |
| Lung infarction | 0 | 1 | 0 | 0 | 1 |
| Pneumoconiosis | 1 | 0 | 0 | 0 | 1 |
| Recurrent bleeding | 0 | 1 | 0 | 0 | 1 |
| Respiratory foreign body | 0 | 0 | 1 | 0 | 1 |
| **Infection** | **2** | **5** | **3** | **0** | **10** |
| Abscess | 2 | 3 | 3 | 0 | 8 |
| Cellulitis | 0 | 1 | 0 | 0 | 1 |
| Odontogenic infection | 0 | 1 | 0 | 0 | 1 |
| **Healthcare-related complications** | **0** | **4** | **2** | **0** | **6** |
| Anastomotic site leakage | 0 | 1 | 1 | 0 | 2 |
| Colon injury | 0 | 0 | 1 | 0 | 1 |
| Disruption of operation wound | 0 | 1 | 0 | 0 | 1 |
| Postoperative bile leakage | 0 | 1 | 0 | 0 | 1 |
| Treated HCC with rupture | 0 | 1 | 0 | 0 | 1 |
| **Hematologic diseases** | **0** | **1** | **1** | **0** | **2** |
| Lymphoma | 0 | 0 | 1 | 0 | 1 |
| Spinal leukemia | 0 | 1 | 0 | 0 | 1 |
| **Immune system diseases** | 0 | **1** | **1** | 0 | **2** |
| Angioedema | 0 | 1 | 0 | 0 | 1 |
| SLE | 0 | 0 | 1 | 0 | 1 |
| **Endocrine or metabolic diseases** | **1** | **0** | **0** | **0** | **1** |
| Sialoadenitis | 1 | 0 | 0 | 0 | 1 |

Data are number of patients applicable to each item. The diagnoses at the index visits were classified according to the modified guidelines from the 11th edition of the International Classification of Diseases.

ICA, internal carotid artery; LA, left atrium; SMA, superior mesenteric artery; HCC, hepatocellular carcinoma; SLE, systemic lupus erythematosus; AOV, ampulla of Vater; RCVS, reversible cerebral vasoconstriction syndrome; AVM, arteriovenous malformations
